# Complete Response of Hereditary Leiomyomatosis and Renal Cell Cancer (HLRCC)-Associated Renal Cell Carcinoma to Pembrolizumab Immunotherapy: A Case Report

**DOI:** 10.3389/fonc.2021.735077

**Published:** 2021-10-15

**Authors:** Tao Wang, Yan Huang, Xing Huang, Zheng Lv, Shuo Tian, Xin Ma, Xu Zhang

**Affiliations:** ^1^ Department of Urology, The Third Medical Centre, Chinese People’s Liberation Army (PLA) General Hospital, Beijing, China; ^2^ Medical School of Chinese People’s Liberation Army (PLA), Beijing, China

**Keywords:** hereditary leiomyomatosis and renal cell cancer (HLRCC), mutation, immunotherapy, follow-up, complete response (CR)

## Abstract

Hereditary leiomyomatosis and renal cell cancer (HLRCC) is a rare autosomal dominant disorder that results from a germline mutation in the fumarate hydratase (*FH*) gene; it manifests as cutaneous leiomyomas, uterine fibroids, and renal cell cancer (RCC). Patients with HLRCC-associated RCC (HLRCC-RCC) have aggressive clinical courses, but there is no standardized therapy for advanced HLRCC-RCC. Here, we describe aggressive HLRCC in a 26-year-old man who presented with RCC that exhibited a novel heterozygous germline insertion mutation in exon 2 of the *FH* gene (c.191dupA: p.N64fs). Systemic lymph node metastasis had already occurred. The patient underwent robot-assisted laparoscopic resection of the right kidney, but new metastases appeared within 5 months postoperatively. Histological staining of the resected tumor showed high expression levels of programmed cell death-ligand 1 (PD-L1) and programmed cell death-1 (PD-1). The patient was treated with anti-PD-1 antibody as first-line therapy. After 2 years of immune checkpoint inhibitor (ICI) treatment, all lesions had disappeared; this response was maintained at 51 months. To our knowledge, this is the first successful treatment of HLRCC-RCC with single-agent immunotherapy. Our approach might be effective for patients with advanced HLRCC-RCC.

## Introduction

Hereditary leiomyomatosis and renal cell cancer (HLRCC) is an autosomal dominant disorder that results from a germline mutation of the fumarate hydratase (*FH*) gene on chromosome 1q42.1 (1, 2). Individuals with *FH* germline mutations are at risk of developing multiple cutaneous and uterine leiomyomas, as well as renal cell carcinoma (RCC) (3). HLRCC-associated RCC (HLRCC-RCC) was defined as a distinct entity in the 2016 World Health Organization classification (4). Importantly, patients with HLRCC-RCC usually have poor clinical courses.

*FH* acts as a tumor suppressor gene that encodes an enzyme in the tricarboxylic acid cycle; this enzyme catalyzes the conversion of fumarate to malate. Intracellular fumarate accumulation leads to the overexpression of hypoxia-inducible factor-1α with resulting pseudohypoxia, which induces angiogenesis and appears to cause tumorigenesis (5).

There are no standard therapies or consensus for advanced HLRCC-RCC. Novel methods (e.g., targeted therapy and immunotherapy) might improve the prognosis of advanced RCC, which would also provide new insights regarding HLRCC. Here, we describe an aggressive HLRCC in a 26-year-old man who exhibited a novel heterozygous germline insertion mutation in exon 2 of the *FH* gene (c.191dupA:p.N64fs). He achieved complete response (CR) to pembrolizumab immunotherapy, an anti-programmed cell death-1 (PD-1) antibody. To our knowledge, this is the first report of pembrolizumab monotherapy producing CR in advanced HLRCC-RCC. The successful outcome in this case may provide new insights for the management of HLRCC.

## Case Presentation

**Figure 1:**
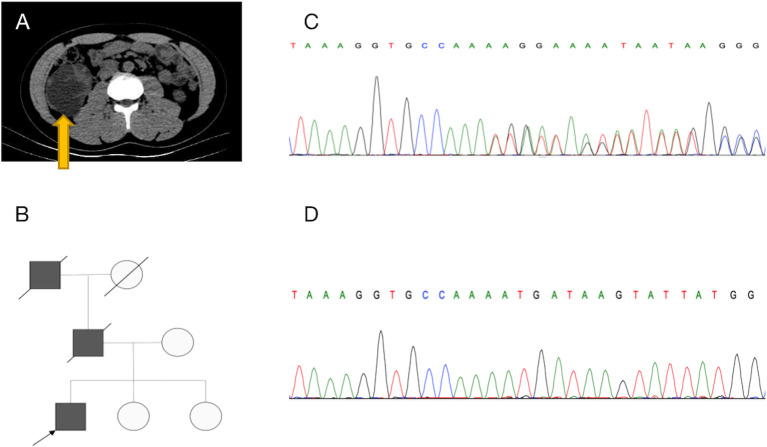
Diagnosis of hereditary leiomyomatosis and renal cell cancer-associated renal cell carcinoma (HLRCC-RCC). **(A)** Transverse CT revealed a large mass in the lower middle part of the right kidney. **(B)** Pedigree of the family with three patients. The black symbols represent the affected members with renal carcinoma, and the arrow indicates the proband. **(C, D)** Genetic testing identified a novel fumarate hydratase (FH) germline mutation (c.191dupA:p.N64fs), which confirmed the diagnosis of HLRCC-RCC. **(C)** A heterozygous mutation was identified in blood, and **(D)** a homozygous mutation was identified in tumor tissue.

The patient was a 26-year-old man with an unremarkable medical history. A painless left supraclavicular lymph node was found incidentally in early 2017; it was considered malignant. Ultrasound-guided biopsy of this left lymph node indicated metastasis of renal adenocarcinoma. Positron emission tomography (PET)-computed tomography (CT) showed that the right kidney volume was increased, the lower part of the right kidney contained an irregular cystic mass, and the internal glucose metabolism was uneven (**Figure 1A** and **Supplementary Figure 2A**). There were multiple enlarged lymph nodes in the neck (zones III–V), bilateral clavicle areas, posterior mediastinum, and posterior phrenic angle space, as well as adjacent to the abdominal aorta and iliac vessels; all of these enlarged lymph nodes were considered malignant (**Figure 3C**).

Because the patient’s father and grandfather both died of kidney cancer (**Figure 1B**), HLRCC was suspected. Whole-exome sequencing of genomic DNA from blood and cancerous tissues was performed after the patient had provided informed consent. The sequencing results revealed a previously unidentified germline insertion mutation in exon 2 of the *FH* gene (c.191dupA:p.N64fs); the mutation rate was strongly enhanced in the tumor tissue. Sanger sequencing confirmed these findings (**Figures 1C, D**) and supported a diagnosis of HLRCC-RCC.

**Figure 2:**
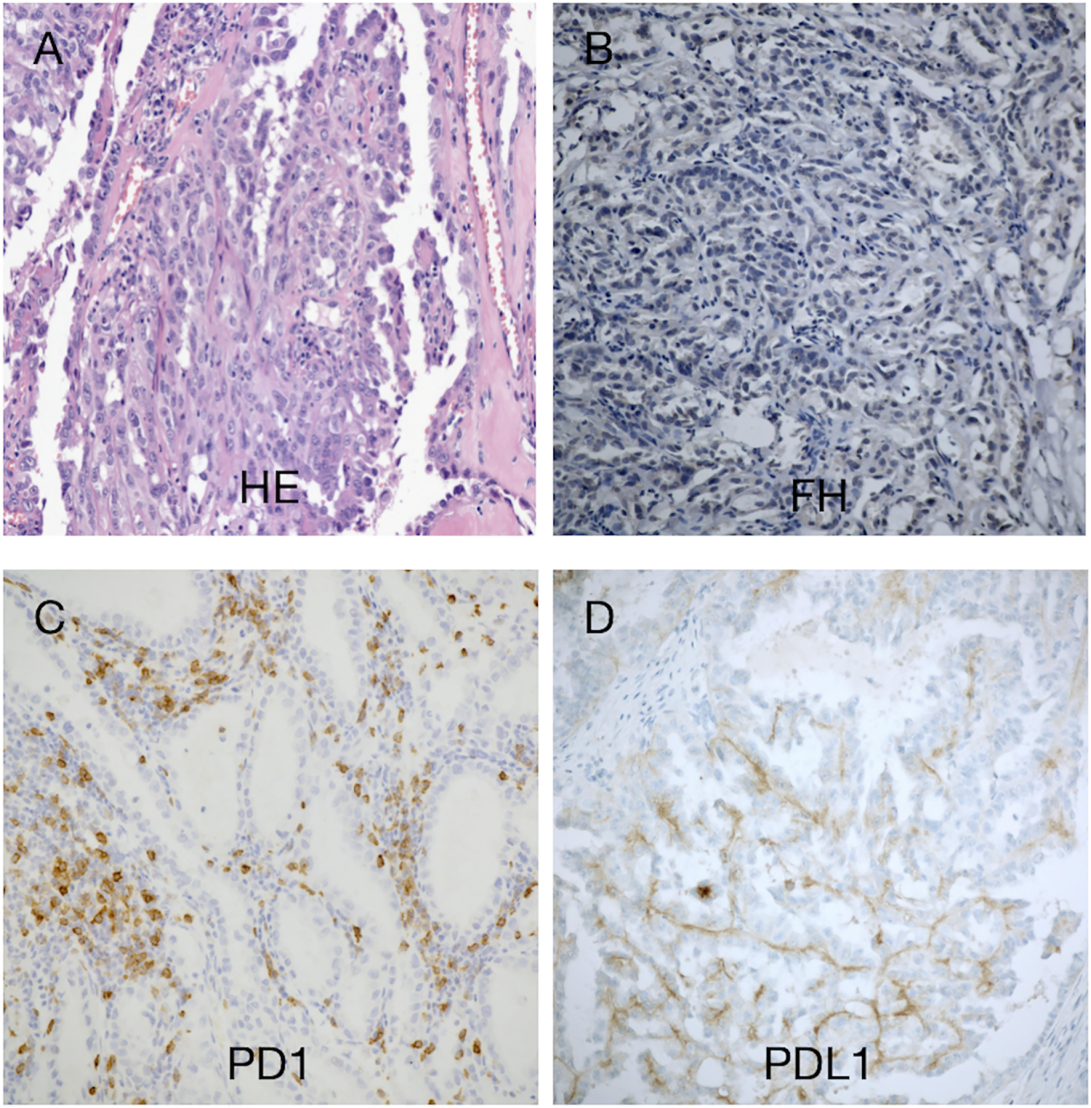
Histomorphological findings in our patient with hereditary leiomyomatosis and renal cell cancer syndrome. **(A)** Hematoxylin–eosin staining showed a renal cell carcinoma with papillary and tubular structures. **(B)** Immunohistochemistry revealed absence of fumarate hydratase (FH) expression in tumor cells, supporting the diagnosis of FH-deficient RCC. **(C, D)** There were few tumor-infiltrating lymphocytes (TILs). Approximately 30% of the tumor cells exhibited programmed cell death-ligand 1 (PD-L1) expression. TILs exhibited programmed cell death-1 (PD-1) expression (35%). Magnification, ×100.

The patient underwent robot-assisted laparoscopic resection of the right kidney and retroperitoneal lymphadenectomy (**Supplementary Figure 2B**). Postoperative pathology revealed RCC with papillary and tubular structures, accompanied by metastasis of the inferior vena cava lymph nodes and invasion of the renal sinus and perinephric fat. The tumor measured 8.5 cm × 6.5 cm × 6 cm and was World Health Organization/International Society of Urologic Pathologists grade III. The microstructure was characterized by thick papillae lined with large tumor cells containing abundant, granular, and eosinophilic cytoplasm; large nuclei were present with prominent eosinophilic nucleoli surrounded by clear halos (**Figure 2A** and **Supplementary Figure 1A**). Immunohistochemistry indicated high expression levels of programmed cell death-ligand 1 (PD-L1) (tumor cells +30%) and PD-1 (lymphocytes +35%) (**Figures 2C, D**). FH staining of the tumor tissue revealed the loss of FH expression (**Figure 2B** and **Supplementary Figure 1B**).

**Figure 3:**
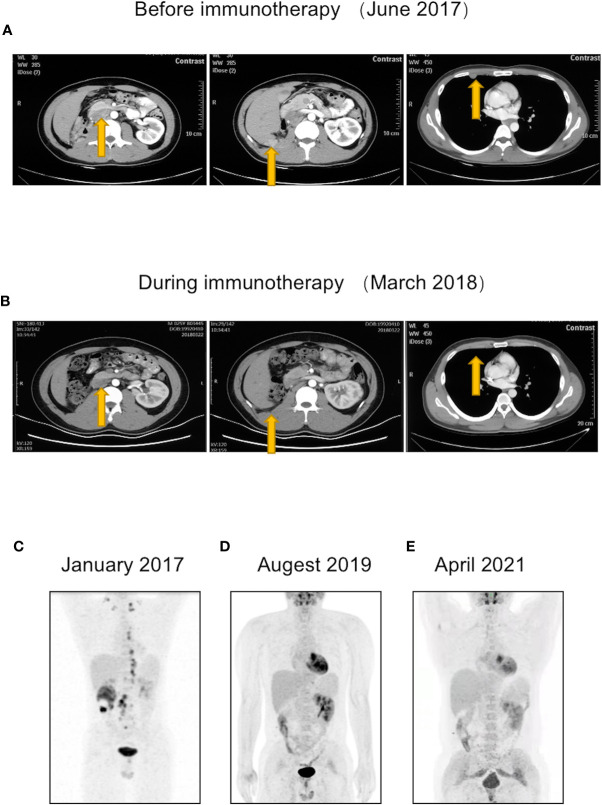
The patient had new metastatic lesions postoperatively but showed a response during immunotherapy and eventually achieved complete response (CR). **(A)** At 4 months postoperatively, CT revealed metastatic lesions on retroperitoneal lymph nodes, peritoneum, and right pleural nodules. **(B)** After 8 months of pembrolizumab treatment, the patient’s abovementioned metastatic lesions were significantly reduced or even disappeared. **(C–E)** Changes in patient’s PET-CT. **(C)** Preoperative PET-CT showed multiple systemic lymph node metastases. **(D)** After 2 years of immunotherapy, PET-CT showed that the metastases had disappeared. **(E)** A third PET-CT in April 2021 confirmed the patient’s CR maintenance status.

**Figure 4:**
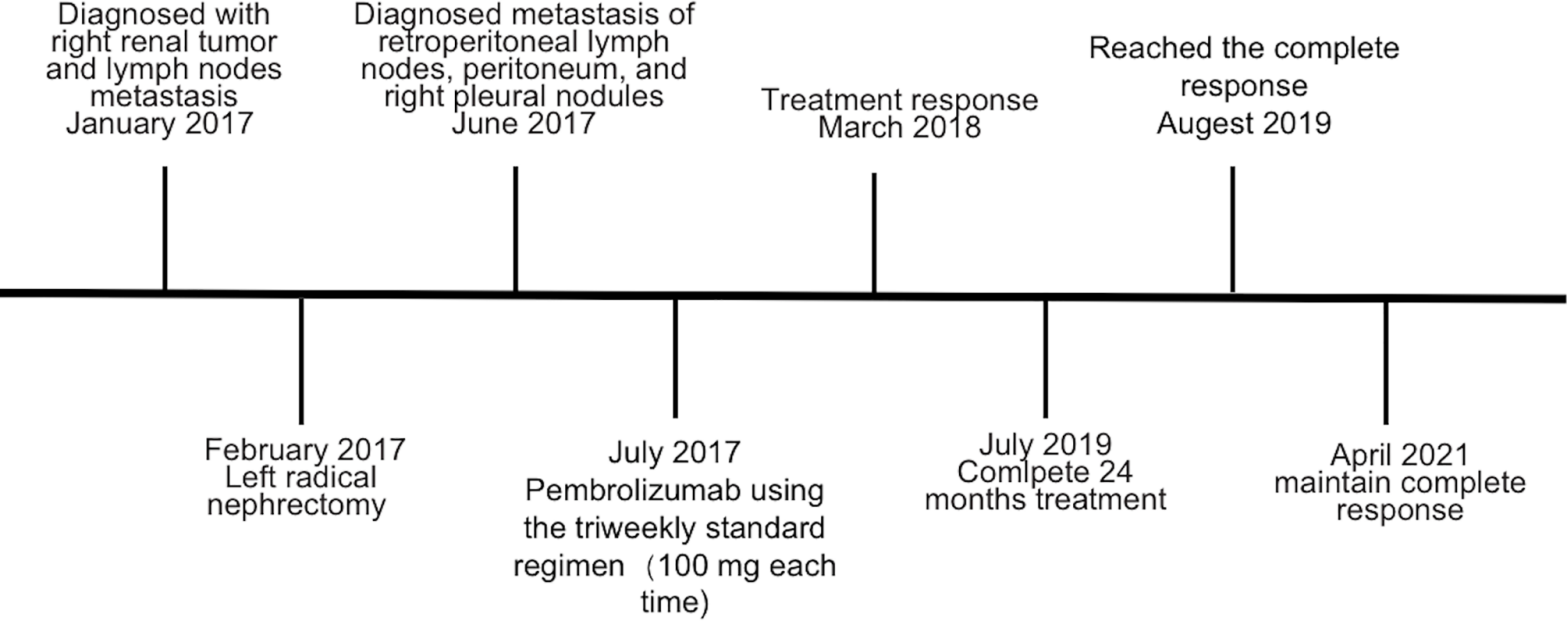
Timeline of the case.

At 4 months postoperatively, CT revealed multiple nodules on the retroperitoneal lymph nodes, peritoneum, and right pleural nodules, suggestive of metastasis (**Figure 3A**). The patient was immediately treated with pembrolizumab using the standard triweekly regimen (100 mg per treatment). The main side effect during treatment was immune enteritis. After 8 months of pembrolizumab treatment, the patient’s abovementioned metastatic lesions were significantly reduced or even disappeared (**Figure 3B**). After 24 months of treatment, PET-CT showed that the fluorodeoxyglucose (FDG) metabolism of the lesions had normalized, indicative of CR (**Figure 3D**). A third PET-CT in April 2021 still showed no disease progression (**Figure 3E**). The timeline is shown in **Figure 4**.

## Discussion

HLRCC is a rare autosomal dominant disorder caused by heterozygous germline mutations in the *FH* gene (1q42.3-43). Approximately 15% of patients with HLRCC develop RCC. Most patients with HLRCC develop aggressive RCC that demonstrates papillary morphology and early metastasis (3). Here, we described a patient with HLRCC-RCC who exhibited a novel heterozygous germline FH mutation and was cured with pembrolizumab. While receiving immune checkpoint inhibitor (ICI) treatment, the patient’s only complaint was mild enteritis. The patient achieved CR after 2 years of pembrolizumab treatment, with improved symptoms and controlled metastasis. To our knowledge, this is the first case of successful single-agent immunotherapy for HLRCC-RCC, providing new insights for the management of HLRCC.

Prompt excision of HLRCC-associated kidney tumors is critical for preventing metastasis (3). However, no standard therapies or consensus management approaches have been established for advanced HLRCC-RCC. The PD-1/PD-L1 axis is currently a therapeutic target for various treatment-resistant neoplasms. Checkpoint inhibitors may also be effective in patients with HLRCC-RCC. There have been several reports of HLRCC-RCC treatment with ICIs, which have attracted attention as a new therapeutic option. Antitumor efficacy has been achieved by targeted therapy and ICI combinations in patients with variant histology RCC (6, 7). A recent study reported the achievement of CR in a patient with HLRCC-RCC after 31 weeks of ICI combination treatment (nivolumab plus ipilimumab) (8). Another study showed that ICI treatment led to improved progression-free survival compared with antiangiogenic monotherapy (9). Several cases of papillary RCC were treated effectively with nivolumab (10–12). A single-arm phase II study of pembrolizumab demonstrated an overall response rate of 25.4% in papillary RCC (13). The Society for Immunotherapy of Cancer also recommended single-agent anti-PD-1 as the first-line treatment for papillary RCC (14).

PD-1/PD-L1 has been shown to improve the prognosis of patients with HLRCC (15). A multicenter phase II study of atezolizumab and bevacizumab for patients with metastatic RCC involving variant histology revealed an overall response rate in PD-L1-positive patients of 60% (n = 9) vs. 19% (n = 4) in PD-L1-negative patients (16). In the report of CR after combined ICI treatment, approximately half of the tumor cells exhibited PD-L1 expression (8). The immunohistochemical staining in our patient showed strong PD-1/PD-L1 expression. Thus, PD-1 and PD-L1 may be useful as predictors or biomarkers of treatment effects in future studies.

The establishment of systemic therapy for HLRCC-RCC is an unmet need. The findings in our case suggest that ICI treatment is an effective therapeutic option, although long-term survival results are not available. Additional cases of immunotherapy in patients with HLRCC should be collected to determine the role of its treatment in HLRCC.

## Conclusions

A novel *FH* gene mutation was found in a patient with HLRCC-RCC. He achieved CR after pembrolizumab monotherapy; this response was maintained at 51 months. Immunotherapy for HLRCC merits further studies in additional patients.

## Data Availability Statement

The datasets presented in this study can be found in online repositories. The names of the repository/repositories and accession number(s) can be found in the article/**Supplementary Material**.

## Ethics Statement

The studies involving human participants were reviewed and approved by Ethical Committee of PLA General Hospital. The patients/participants provided their written informed consent to participate in this study.

## Author Contributions

TW and YH: performed the research and wrote the article. XH and ZL: performed the experiments and collected patient data. ST: assisted with laboratory experiments and produced radiology images. XM and XZ: contributed to patient samples and treated patients. All authors contributed to the article and approved the submitted version.

## Funding

The study was supported by the National Natural Science Foundation of China (Grand Nos. 81770790 and 81970665).

## Conflict of Interest

The authors declare that the research was conducted in the absence of any commercial or financial relationships that could be construed as a potential conflict of interest.

## Publisher’s Note

All claims expressed in this article are solely those of the authors and do not necessarily represent those of their affiliated organizations, or those of the publisher, the editors and the reviewers. Any product that may be evaluated in this article, or claim that may be made by its manufacturer, is not guaranteed or endorsed by the publisher.

## Supplementary Material

Supplementary Figure 1 Magnifying Hematoxylin–eosin image and the internal control of FH. **(A)** Hematoxylin–eosin staining showed the prominent nucleoli and perinucleolar halo, which is so characteristic of this entity.Magnification 400× **(B)** *FH *staining showed positive in the tissues of FH wild-type patients, proving the effectiveness of our immunohistochemistry process.Magnification 100×.

Supplementary Figure 2 Additional computed tomography information. **(A)** Axial images at different levels before operation. **(B)** Images at post-operative baseline.
