# Supplementary figures and images for: Complete Response of Hereditary Leiomyomatosis and Renal Cell Cancer (HLRCC)-Associated Renal Cell Carcinoma to Pembrolizumab Immunotherapy: A Case Report
Source: Front Oncol. 2021 Oct 15;11:735077. doi: 10.3389/fonc.2021.735077 (PMC8554149; doi:10.3389/fonc.2021.735077)

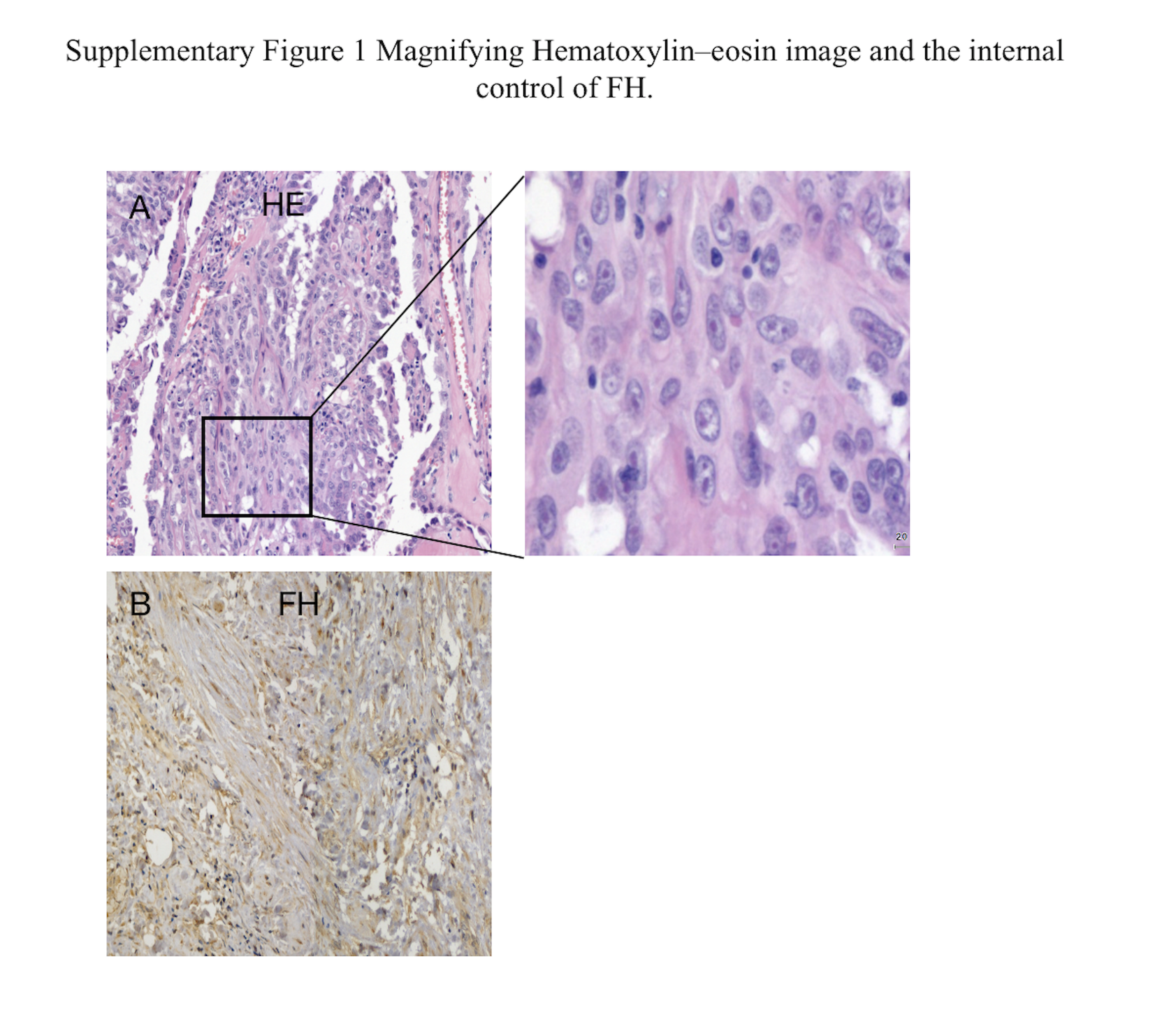

Supplement: Supplementary Figure 1 — Magnifying Hematoxylin–eosin image and the internal control of FH. (A) Hematoxylin–eosin staining showed the prominent nucleoli and perinucleolar halo, which is so characteristic of this entity.Magnification 400× (B) FH staining showed positive in the tissues of FH wild-type patients, proving the effectiveness of our immunohistochemistry process.Magnification 100×. [file Image_1.jpeg]

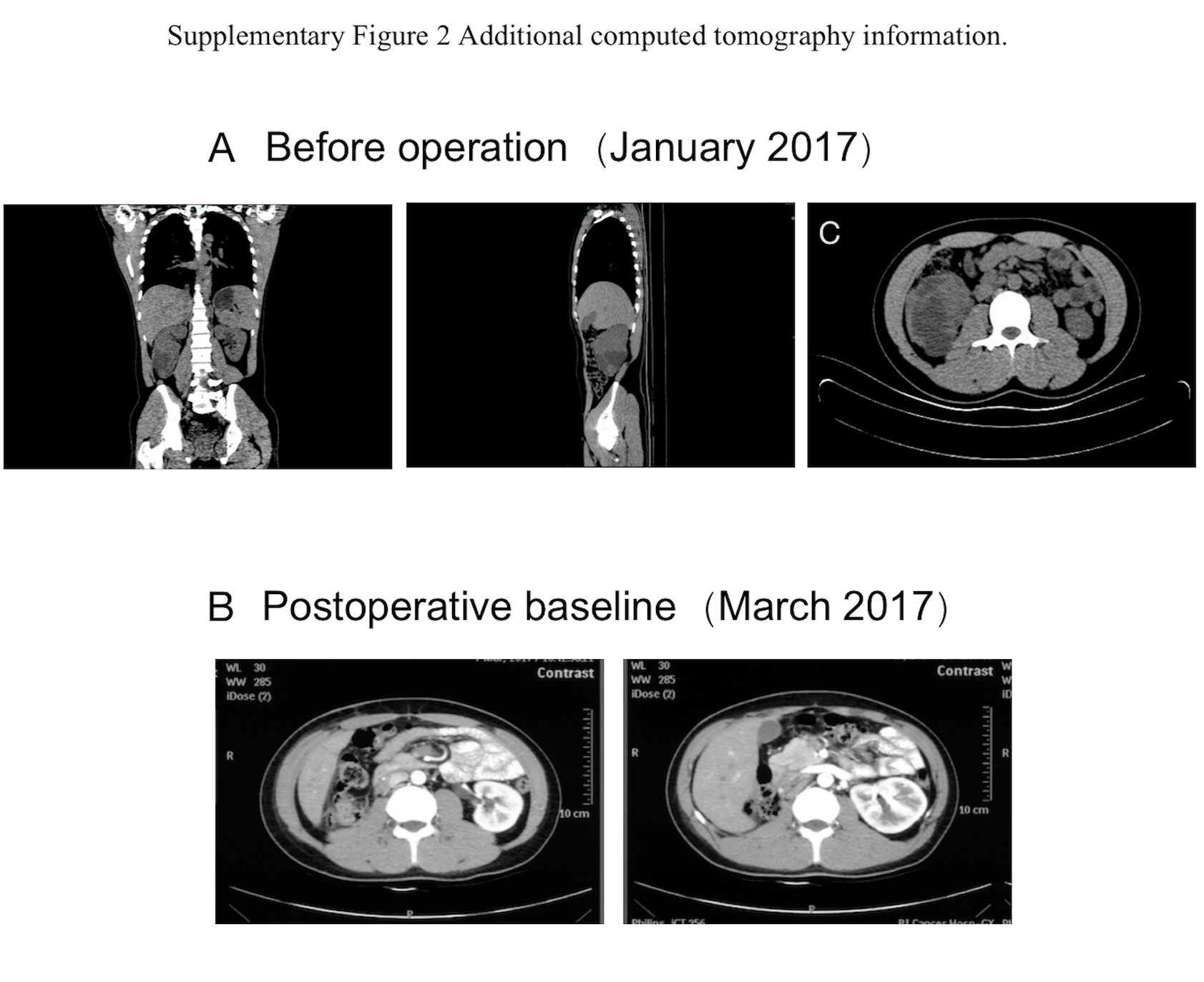

Supplement: Supplementary Figure 2 — Additional computed tomography information. (A) Axial images at different levels before operation. (B) Images at post-operative baseline. [file Image_2.jpeg]
